# On Physical Diagnosis in Fevers

**Published:** 1855-01

**Authors:** E. A. Pakkes

**Affiliations:** M. B. Lond., L. R. C. P., Professor of Clinical Medicine, University College, London


### RECORD OF MEDICAL SCIENCE.

*On Physical Diagnosis in Fevers.* By E. A. PAKKES, M.B. Lond., L. R. C. P., Professor of Clinical Medicine, University College, London.

*Typhus and Typhoid.*—Dr. Parkes, last week, after describing all the familiar symptoms of typhoid and typhus, went into considerable length as to the difficulties of diagnosis. Occasionally we have local manifestations of disease of such severity (he observed) as may lead us to overlook the nature of the disease altogether. We may treat cough, dyspnœa, bronchitis, in a word, with all its various phases, and yet the disease be typhoid fever; other times, diarrhœa of a most troublesome kind is persistent—indeed many deaths are registered in London as deaths from diarrhœa, but they are nothing more or less than deaths from typhoid fever. Yet, if a correct examination were made, and the history of these cases made out, they would give us the tenderness of the iliac fossa, the rose-spots I have just described as so characteristic of typhoid, and in fact all the progressive conditions of this diseate. Again, we may have nervous symptoms predominant—an ataxic form of typhoid; and here you will find very great difficulty indeed in the diagnosis. These difficulties are not attended to sufficiently in practice.

Fourthly, we may have a still more troublesome and insidious form of typhoid, attended with weakness and weariness, excessive thirst, no shivering; the patient has no very marked symptoms of any kind, yet suddenly dies of perforation of the intestine. All these difficulties of diagnosis should lead us more and more to study the disease, as only by seeing the entire features of the history of the case, can we come to understand really what it is.

We will now, as illustrations of typhoid, take one or two of the cases up stairs in the wards at present, and I will read to you from the case-book the history:—

T—— ——, aged 21, admitted September 30. Her previous history, as in all such cases, is a little deficient, as when patients are very bad in fever you cannot get that connected account you wish. She was born of phthisical parents; she has been very poor, and has suffered many privations. The disease first came on by vertigo, shivering, and, after the fourth day, profuse purging, with extreme weakness, loss of appetite, thirst; two or three days after, pain in the abdomen. Diarrhœa lasted fully a week, headache also continued for that time; muscular weakness and thirst not abated. She then sought relief in University College Hospital. (Dr. Jenner, who has described this disease, also saw her; these cases, in fact, are now very interesting.) The symptoms in hospital were first, on coming in, excessive heat and dryness of skin, as shown by the thermometer at 105° Fah.; flushed face; abdomen presenting the peculiar red-rose spots, disappearing on pressure, slightly elevated; in fact, the rose-spots we now recognize as so characteristic of this disease in contradistinction to the mulberry-colored blotches of typhus. She complained of frontal headache, with other nervous symptoms well-marked; tightness across the sternum, frequent cough, and, on stethoscopic examination, dry bronchitis; but no deeper-seated disease of either lungs, or chest generally; the actions of the heart quite normal; the pulse, I should have said, so high as 116. Careful palpation over the liver detected nothing wrong; the same remark applies to the opposite side, at the angle of the large intestine and spleen. She had, as many of you will remember (I dwelt upon it at the time,) excessive tenderness over the abdomen, and more especially in the right iliac fossa; there was marked anorexia; the stools were liquid, yellowish, granular; the tongue large, and red at the tip, moist, not at all the tongue of typhus. You will remember, we have seen all these symptoms in more than one other case to-day also, as we were going round; they are very worthy of study, as so often met in medical practice.

As this subject of physical diagnosis in fevers is one in which I wish to exercise you, as it is, in fact, in practice in England, one of very eminent usefulness, and, in London, a matter in which at any moment you may be called upon to put in force, we will skip now from this part of the case, and come to the sixteenth day of the disease. And how are you to know it? You are brought to see this young woman, we will say, for the first time; the specific rose-spots are gone; she is laboring to all intents and purposes under severe bronchitic and chest symptoms (a chemist, or practitioner with a druggist's shop, has prescribed, and given cough mixtures perhaps, without seeing her); you find her respiration 30 in a minute, cough incessant, with some expectoration, nervous symptoms also well-marked; vertigo complained of, torpor, the eyes closed; she is delirious at night; she has also diarrhœa, pain over the abdomen, pulse quick, tongue furrowed and somewhat coated. Suppose, I say, you were called to such a patient, and moreover she is unable to give any account of the previous illness, how are you to make the diagnosis? There are only two ways—one the positive method, the other the method as it is called by "exclusion." The first is obvious enough, and will of course be more valuable to the practised eye of the experienced physician, who seizes the nature of the case at the first glance by a sort of intuitive knowledge of what typhoid really is. Now the method of diagnosis by exclusion—the plan of logic-writers, *per viam exclusionis*, in this and other diseases, is one, though not without disadvantages, yet of no mean importance. The first question you resolve in your mind will be—Is she or he, as the case may be, laboring under any of the idiopathic fevers? any of the exanthemata? No. Is it typhus? You make the same answer, as the eruption in ty-*phus* is as different from ty-*phoid* as scarlatina from measles. The eruption is absent in patients under 22 or 21 (this patient's age is about this.) Is it relapsing fever, so common in some years, as 1828–29? No. You ask yourself then, is it typhoid? Yes, nervous symptoms are marked, chest symptoms and diarrhœa also; the latter loose, granular, yellow, so peculiar to typhoid. You have soreness of the right iliac fossa; but then you say we have no rose-spots, and then you remember in at least 20 per cent. these rose-spots are not found. You must weigh and balance all these circumstances in your mind.

There are two diseases not unlike typhoid, to which I wish now to direct your attention, and which may be mistaken, and are mistaken for typhoid—one is *pyæmia*, so called, but it is quite untrue there is any pus in the blood; the other is *acute tuberculosis*. In pyæmia we have its positive indications absent, such as inflamed joints, diseased veins, &c.; we have septic materials in the blood in pyæmia, and a vital change perhaps in that fluid, but you cannot well mistake it for typhoid fever. There is another disease, however, which has been lately quite mistaken for typhoid—this is *acute tuberculosis*, in which, more or less, every organ in the body becomes studded with tubercles, and known in England and the Continent as "miliary tubercles," in the intestines, heart, lungs, and in the female, even in the uterus, and pelvic viscera. It is a disease common in younger patients. The disease, however, is extremely rapid, in three or four weeks usually coming to an end. It is attended with febrile symptoms, furred dry tongue; the symptoms, in fact, all like as possible of typhoid. It runs parallel, so to speak, with typhoid, but is not typhoid. Acute tuberculosis is often mistaken for typhoid, but the *rose-spots are absent*. In these cases the best observers will make mistakes. [We rather think the Editor of the *Medico-Chirurgical* here alluded to Dr. Lyons of Dublin and Dr. Stokes.] These tubercular deposits are miliary; they are uniform over the lung. We have no opportunity of comparing disease in one part of the lung with another; no stethoscopic indications, in fact, but those of bronchitis. Again, in this disease of acute tuberculosis, the head symptoms are always most intense, from deposit, in the shape of acute meningitis; the latter produced by deposit of tubercle. It is, in fact, something quite out of the common to find bad headache in typhoid; torpor is more common; and according as the disease advances, as a general rule, head symptoms are found to go away. You will find, also, if you study these cases in the wards of the hospital for yourself, that the pupils are dilated in meningitis, and that the special senses of hearing, taste, smell, &c., are all more or less affected. Deafness, for instance, is common; and, as I have just said, you will have most intense headache. Again, in one disease the abdominal symptoms will seize your attention; in the other, the head or chest symptoms. Diarrhœa is not so frequent in one; while in the other it is almost specific. In acute tuberculosis, you will not find the intestinal glands red and inflamed; in the other affection we are speaking of, it is very characteristic after death. The typhoid stools are liquid, yellow, or brown, containing albumen, and coloring matter of bile; this singular substance rendered mahogany-color by nitric acid. In hospitals I would advise you to familiarize yourselves with all these circumstances.

To sum up the whole matter, then, you will find that in these two diseases, confounded by superficial observers, we have a prominence, at the bedside, of head symptoms in one, and abdominal symptoms in the other. We have symptoms also of pyrexia in one. We may have "typhoid pneumonia," using these words now in a different sense, "typhoid" as an adjective; with all the other differential signs indicated. Then bad influenza, with capillary bronchitis, may also be mistaken for one of these diseases, purulent meningitis and a disease lately described in Ireland "cerebro-spinal meningitis."—*Dublin Medical Press.*

